# Health professional perspectives on translation of cultural safety concepts into practice: A scoping study

**DOI:** 10.3389/fresc.2022.891571

**Published:** 2022-07-28

**Authors:** Lynere Wilson, Amanda Wilkinson, Kelly Tikao

**Affiliations:** Centre for Postgraduate Nursing Studies, University of Otago, Christchurch, New Zealand

**Keywords:** attitude of health personnel, cultural bias, cultural competency, cultural safety, clinical practice, theory-practice relationship

## Abstract

People from unique and diverse populations, (i.e., social groupings excluded by the dominant majority by, for example, ethnicity, gender, age, sexual orientation, disability or even rurality), experience dissimilar health outcomes. Members of such populations who have long-term health conditions experience further health disparities through inefficient management and treatment. This remains a significant hindrance to achieving equity in health outcomes. Being responsive and acting upon the cultural needs of unique and diverse populations within health services is pivotal in addressing health disparities. Despite provision of professional training to health professionals, cultural competency remains an elusive goal. This scoping study summarized available literature about what helped health professionals translate cultural safety concepts into practice. We searched electronic databases using MeSH terms and keywords for English language articles and reference lists of potentially included studies. Quality appraisal was undertaken using Joanna Briggs Institute critical appraisal tools. Data were charted, with a descriptive numerical summary and thematic analysis of study findings undertaken. Twelve qualitative studies with n = 206 participants were included. Learning through and from direct experience, and the individual qualities of professionals (i.e., individual capacity for relational skills and intentionality of engagement with one's own values and biases) facilitated translation of cultural safety concepts into practice. Also important was the need for cultural training interventions to address both issues of content and process within course design. Doing this would take into consideration the benefits that can come from learning as a part of a collective. In each of these themes was evidence of how health professionals needed the ability to manage emotional discomfort as part of the process of learning. A dearth of information exists exploring professionals' perspectives on translating cultural safety concepts into practice. There may be merit in designing educational interventions that look beyond the classroom. We also suggest that nurturing people's relational skills likely holds benefits to growing culturally safe practice as does increasing health professional's capacity to sit with the discomfort that occurs when paying attention to one's own and others values and biases.

## Introduction

People from unique and diverse populations, that is social groupings who are excluded from the dominant majority by for example, ethnicity, gender, age, sexual orientation or geography, are likely to experience dissimilar health outcomes to the majority population (1–4). Members of such populations who have long-term health conditions experience further health disparities through inefficient management and treatment (5). These factors remain a significant hindrance to achieving equity in health outcomes. Being responsive to the cultural needs of unique and diverse populations within health services is pivotal in addressing health disparities (1, 6).

The capacity to provide health care that is responsive to cultural factors impacting on health and wellbeing has been given various labels; cultural awareness, cultural sensitivity, cultural competence and cultural safety, with each one meaning something slightly different. In line with Curtis et al. (7) we have taken the stance that notions of “awareness” and “competence” promote a focus upon the knowledge, skills and attitudes of the individual health professional as if all they need is know the customs of the “exotic other.” Competence also suggests a static object, the presence of which can be successfully measured such that once held, it is never lost. Instead we take the position that cultural safety, with a genealogy to the New Zealand nursing context, is a more useful construct for all health professionals.

Cultural safety as a concept was developed by Dr. Irihapeti Ramsden in 1990, and in 1992 the New Zealand Nursing Council commissioned a set of guidelines for how to incorporate cultural safety within nursing and midwifery training. The objectives were to educate nurses and midwives to examine their own culturally based values and beliefs, to develop the capacity to be opened minded and flexible toward people of cultures other than their own, to have nurses and midwives who recognized the impact of social and historical processes upon a person's current situation and to produce a workforce of people who were deemed to be culturally safe as defined by the people using the health service (8). In a colonized country such as New Zealand, with a history of broken promises to Māori (made in our founding document Te Tiriti o Waitangi), cultural safety remains highly relevant. Cultural safety as a concept is not without its critics, with some arguing that its development within a bicultural context does not make it usefully transferable to notions of multiculturalism (9). However, with its focus on the expression of power in relationships and the development of self-awareness within the health professional of their own values and how these play out in the health care relationship, we take the position that cultural safety is a concept that is both productive and transferrable.

Despite the provision of culturally focused training and professional development, having a workforce of culturally capable health professionals remains an elusive goal (10–14). While educational interventions offer much as a potential means to improve culturally competent practice, they are not the panacea that had been hoped. Reviews of the efficacy of education interventions for health professionals have found similar outcomes; that it can change individual health professional's level of knowledge and confidence but it is unclear if it changes practice such that patients experience better health outcomes as a result. Methodological problems with studies (not defining what is meant by culture and competency, no objective measure of change in practice and little attempt to define the core elements of training that bring about change in practice) mean that there is little to guide the development of effective educational initiatives (12, 13, 15, 16).

As identified by these reviews of educational interventions, one of the methodological issues with research in the area is that little attention is often paid to what is meant by the term “culture.” For those that study “culture” i.e., anthropological based studies, it is a contested construct with each preferred definition reflecting different philosophical stances (17). The stance taken for this scoping review is that culture is multi-faceted; it is the beliefs, values, ways of making sense of the world that people inherit. For some this inheritance comes from growing up in a culture. For others it is an inheritance through whakapapa (genealogy) as the effect of colonization has been to disconnect them from their culture. Culture is therefore dynamic and ever changing in response to social conflicts, migrations and other forms of social transformation (18). Culture is also the daily social practices of individuals and small groups and the enactment of shared life experiences, “those *shared local world of interpersonal experience*–neighborhood, villages, social networks–where culture is enacted through processes of social interaction…” [(19) emphasis in original]. Arguably, these attempts to define “culture” still miss the mark as they remove the people themselves and discuss values and beliefs as components of a culture rather than culture as the people who live, eat, pray and actually are these values. These attempts to define “culture” can also be seen as “otherising” or placing a definition upon something not someone. Identity, values and beliefs can appear flippant in descriptions of culture, as components of a culture rather than capturing the living culture. Culture therefore is more than ethnicity, race, country of birth or religion. It is the many different social groupings of which people are part of on the basis of shared experiences of gender, sexuality, age, social class and the numerous different ways that people might use to group themselves. It is the ways of thinking that a person uses to make sense of the world and their place in it. It is all the small, daily practices through which a person enacts their values and beliefs and which in turn shapes identity. When these practices of being a human are diminished by or not recognized by others, then so too is the person. Culturally safe practice therefore seeks to uphold the integrity of the person and the communities of which they are part.

With the current limited information of the most effective ways to develop culturally safe practice in health professionals, we have instead chosen to explore this conundrum from the perspective of health professionals themselves. While Graham and Masters-Awatere (10) argue that in a New Zealand context, culturally safe health professionals would seem to be their own minority group, there is still much we could learn from those who are. We have therefore taken an overtly strengths-based approach by wanting to understand the perspectives of health professionals about what has helped them to translate ideas about cultural safety into their practice. We argue that if healthcare professionals are not culturally competent and safe in their practice, how can they effectively support self-management endeavors of people in unique and diverse populations. Therefore, this review aimed to scope and summarize available literature on healthcare professional perspectives on what facilitates the translation of cultural safety concepts into practice.

## Methods

The review was guided by Levac et al. (20) six stages for conducting a systematic scoping review.

### Stage one: Identifying the research question

The aim of the study was to scope and summarize available literature on healthcare professional perspectives on what helps and hinders the translation of cultural safety concepts into practice.

### Stage two: Identifying relevant articles

An iterative and systematic search of databases (Proquest, CINAHL, Scopus, Medline, Embase, Emcare, PsycInfo) was conducted up to June 2021 using MeSH and keywords for peer reviewed, English language articles. MeSH subject headings of “cultural competence,” and “cultural diversity” and “attitude of health personnel” were used. In addition, keyword phrases of “cultural safety,” “cultural bias,” “theory-practice relationship,” “knowledge transfer and health care” “critical reflection,” “unconscious bias,” “health professionals” and “barriers to implementation” were used (see Table 1).

**Table 1:** MeSH and keywords used.

**MeSH**	**Keywords**
Cultural competency	Cultural safety
Cultural diversity	Cultural bias
Attitude of health personnel	Theory to practice relationship
	Knowledge transfer
	Health professional
	Critical reflection
	Barriers to implementation

**Figure 1:**
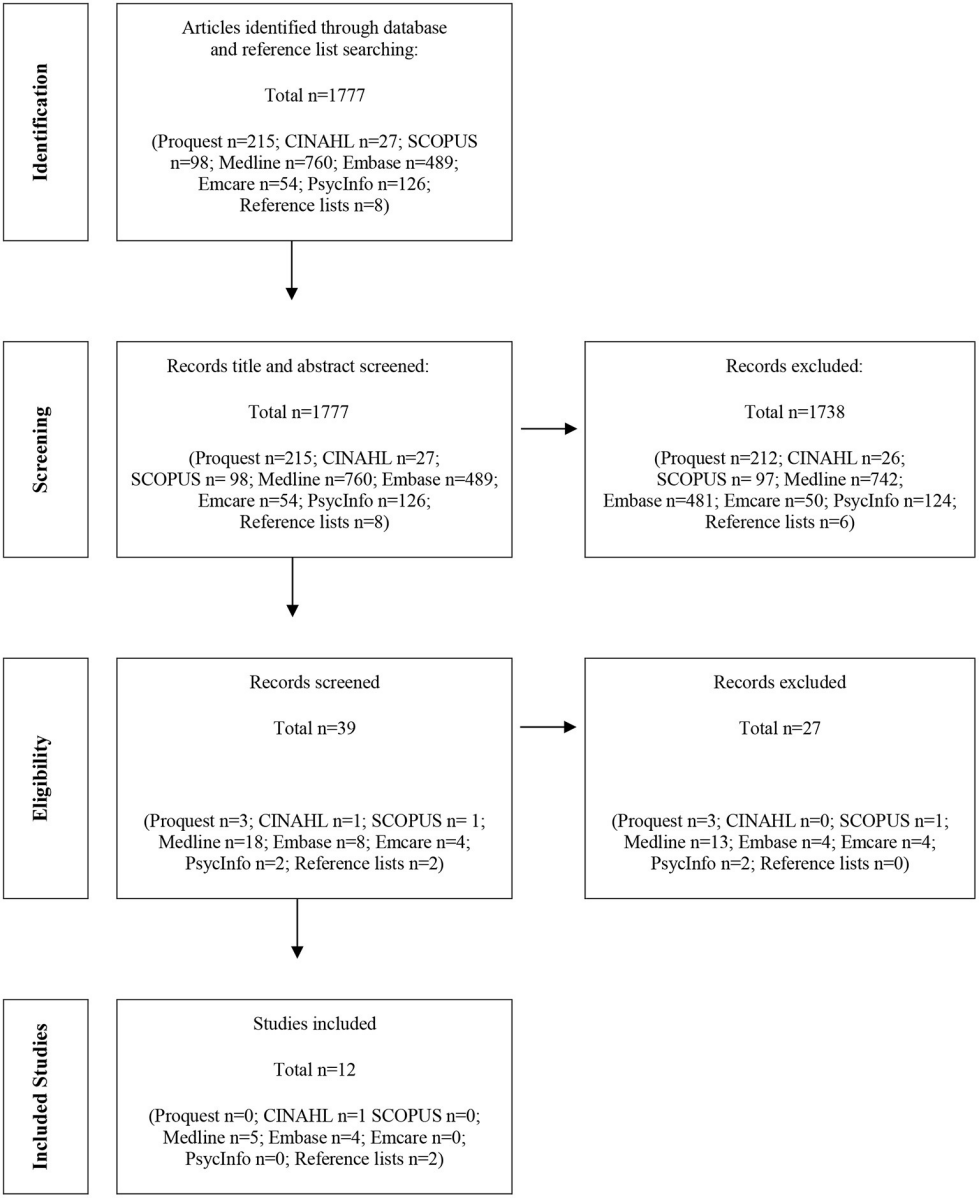
PRISMA flow diagram of articles through the study (21).

One author (LW) title and abstract screened potential articles and included them if they met the inclusion criteria. The inclusion criteria were set as broadly as possible as it became apparent early in the process that there was likely limited literature that directly answered the research question. Articles needed to be in English and include data that was likely to help answer the research question. No date limits were set. Those that were unclear were discussed with a second author (AW). Reference lists of potentially included studies were also searched for further potential articles. Figure 1 provides a summary of the flow of articles through the review (21).

### Selection of relevant studies

Scoping reviews do not usually require a quality appraisal of included studies. For this review, we chose to do so as much of the work of searching and screening had been undertaken by one author (LW). Methodological quality of included studies was undertaken using appropriate JBI critical appraisal tools (22). Quality for inclusion was deemed to be met if 70% of the JBI quality criteria were met. One author (LW) quality appraised all studies, with the second author (AW) quality appraising a random selection of 20% of the studies. Both authors then discussed their appraisal findings together. While there were no disagreements regarding the inclusion or exclusion of studies, this was a process that assisted the iterative process of reviewing the search strategy and inclusion criteria to ensure a breadth of data for analysis.

### Stage four: Charting the data

Data pertaining to author, year, country, setting, aim, method, participant number and description, cultural safety definition and findings were charted (Table 2). A descriptive numerical summary was undertaken for information pertaining to country, participant number and description, and cultural safety definition. Guided by Braun and Clarke's (23) approach to thematic analysis, the initial familiarization stage was followed by extracting all the direct quotes that were relevant to this review, and development of initial codes. Initial themes were then selected and further refined through numerous discussions between authors (LW and AW). Themes were then named and described. Writing the findings section for this article provided further opportunity for refinement of the themes with the third author (KT).

**Table 2:** Summary of included studies by author, year, country, setting, aim, method, participants, cultural safety definition and findings.

**Reference**	**Country, setting**	**Aim, method**	**Participants**	**Definition of cultural safety**	**Findings**
Blanchet Garneau et al. (32)	Canada Three publicly funded urban health and social services organizations	Examined interactions between participants and the larger structures of healthcare systems impacting on development of cultural competence Grounded theory–participant observation (29 hrs) and semi-structured interviews	Total *n* = 24; F *n* = 24; *n* = 13 registered nurses and *n* = 11 undergraduate nursing students Age range nurses 28–59 yrs, students 20–47 yrs Ethnicity *n* = 10 nurses and *n* = 6 students Canadian, *n* = 5 students and *n* = 3 nurses outside of Canada	Not stated–refers to cultural competence, transcultural nursing (51), the person-environment (52) and cultural competence from an organizational perspective	Two concurrent processes identified–(i) dealing with structural constraints and (ii) mobilizing social resources
Castell et al. (35)	Western Australia Large university	Explored the level, nature, and content of critical reflexivity engaged in by students undertaking a unit in Indigenous and cross-cultural psychology. Qualitative approach-reflexive writing exercise	Students enrolled in 3^rd^ yr undergraduate psychology unit; T1 start of unit: Total *n* = 44; F *n* = 26; Male trans *n* = 1 T2 end of unit: total *n* = 23; F *n* = 16 Age range 18–25 yrs Ethnicity listed as Australian, Asian, British, Irish, American, South African and African	Not stated–refers to decolonising the curriculum through transformative learning theory (53) and critical reflexivity.	Time 1: Statements were at Habitual/Action and Understanding levels Time 2: Statements at Understanding and Reflection levels Used Kember et al. (54) four-category scheme for thinking to code data: 1. Habitual Action/non-reflection (no attempt at understanding the topic) 2. Understanding (with no application of knowledge) 3. Understanding (with no application of knowledge) 4. Reflection (application of topical knowledge in relation to personal experience or self) 5. Critical reflection (with engagement in examination of, and shifts in foundational assumptions)
Downing and Kowal (31)	Darwin, Australia Hospital based nurses caring for Aboriginal and Torres Strait Islander people	Explored the role and impact of cultural safety training had on practice Qualitative research methodologies–extensive semi-structured interviews	Total *n* = 6; F *n* = 6; nurses with half trained overseas. Age not stated Ethnicity not stated	Not stated-refers to Indigenous cultural training aimed at assisting healthcare workers to provide “culturally safe health services”	Four themes identified. There is a role for cultural training to assist nurses to: 1. Address racism and provide education about Australian history 2. Hear about differences in communication styles 3. Identify limitations and barriers encountered when putting cultural training into practice, and 4. Identify their personal experience and pre-existing attitudes and how these impact on their response to cultural training
Gray and McPherson (24)	New Zealand Public register of occupational therapists and documents	Reported participants' attitudes to cultural safety Qualitative research paradigm-semi-structured interviews and document analysis	Total *n* = 13; F *n* = 13; Registered occupational therapists registered pre 1993 (*n* = 7) and post 1993 (*n* = 6) Age range 21–65 yrs Ethnicity *n* = 11 European, *n* = 2 European/Māori, *n* = 1 English, *n* = 1 Dutch Cultural safety (from NZ occupational therapy schools), cultural competence and ethics documents (from professional body)	Cultural safety is “about positive attitudinal change toward those who are culturally different from ourselves and learning about power relationships between health professionals and clients. In turn, this attitudinal change and learning is intended to enable occupational therapists to offer a more appropriate and effective service to clients from diverse cultures. When viewed this way, Cultural Safety has a scope that can lead to it being useful for all client/ health professional relationships, regardless of ethnicity (55, 56).	Four themes identified in the data: 1. Generational differences (perceptions, education and knowledge) 2. Personal experiences (more important than academic learning) 3. Attitudinal ambiguity (supported some issues and disagreed with others) 4. Conceptual confusion (about theoretical and practical meaning for some terms) Used continuum of cultural safety from Nursing Council NZ documentation. Awareness–self-knowledge–understanding and practice wisdom to guide analysis.

Hunter and Cook (26)	New Zealand Direct care in primary or secondary health services	Provided insight into participants' holistic indigenous world view and contextualize their professional practice experiences. Qualitative indigenous narrative inquiry	Total *n* = 12; F *n* = 12; Nurses (*n* = 10) and nurse practitioners (*n* = 2), Age range 25–53 yrs Ethnicity self-identified as Māori	Uses Woods (57) a socioethical model of three concentric circles. Innermost–*culturally safe practice*, outermost two social elements of *synthesizing sociocultural practices* and *using socio-political dynamics*, allied with moral elements of *operating within a socially oriented ethic*.	Four themes: 1. Clinical care was prioritized over cultural needs (which are integral to care for Māori) 2. Critical reflection about broader equity issues is needed by non-indigenous nurses to provide ethical care 3. Need to retain indigenous nurses as they contribute to positive healthcare outcomes *via* their advocacy and cultural “fit.” 4. Culturally safe care required committed leadership
McGough et al. (30)	Perth, Western Australia Mainstream mental health services	Described the experiences of mental health professionals caring for Aboriginal people Grounded theory–semi structured interviews, field observations, memo writing, and reflective journaling	Total *n* = 28; F *n* = 17; Registered nurses (*n* = 25) and psychologists (*n* = 3) Age range 25–>61 yrs Ethnicity *n* = 12 Australian born, *n* = 10 UK, *n* = 6 other overseas countries	Not defined. Refers to “Both professionals and institutions work to establish a safe place for patients, which is sensitive and responsive to their social, political, linguistic, economic and spiritual concerns (9). It is more than an understanding of a patient's ethnic background (58) as it requires the ‘health professional to reflect on their own cultural identity and on their relative power as a health provider” (59) *(p. 12)*.	A theory was developed explaining the process used by healthcare professionals when providing culturally safe mental healthcare for Aboriginal people. Healthcare professionals had limited understanding about the concept of cultural safety. They felt unprepared (ill equipped, powerless, and confronted). The cultural safety journey disrupted their self-awareness (they felt they had limited experience, skills and knowledge; felt overwhelmed, and unprepared by the system), and their emotions fluctuated (fearful, anxious, sad, shamed, guilty, felt defeated). Healthcare professionals neutralized the differences (avoided, denied the need to change and minimized the differences), but then with gained confidence, sought new solutions (sought education, reached out to peers, worked with Aboriginal liaison officers).

Molloy et al. (29)	Australia Public mental health services (acute inpatient, community and emergency services)	Explored culture of mental health nursing practice when caring for Aboriginal and Torres Strait Islander service users Multi-sited ethnographic inquiry Interviews and observational fieldwork	Interviews–Total *n* = 17 Fieldwork–*n* = 2 non-participant observations (regional and inner-city service); nurses Age not stated Ethnicity not stated	Provision of care that is holistic, free of bias and racism, challenges belief based upon assumption and is culturally safe and respectful. It is about the person who is providing care reflecting on their own assumptions and culture in order to work in a genuine partnership (60) (*p. 9)*.	Mental health nurses viewed their role as “specialist practice” within the delivery of mental healthcare for Aboriginal and Torres Strait Islander peoples. Despite attending mandatory training in Indigenous health, mental health nurses were unclear what specialist mental health practice consisted of, highlighted gaps in their own knowledge about the culture of the people, and were not confident in the care they provided.
Nielsen et al. (33)	Denmark, University hospital	Explored whether the ethnic patient coordinator program had an impact on health professionals' self-assessed competences during an encounter with patients of ethnic minority. Hermeneutic phenomenological approach-field observations and individual interviews (*n* = 7)	Total *n* = 30 active coordinators; F = 27; *n* = 22 nurses, *n* = 3 midwives, *n* = 2 dieticians, *n* = 1 each of social worker, interpreter and doctor Age not stated Ethnicity not stated	Not defined. Refers to cultural competency (61).	Three main themes identified: 1. Recognizing language barriers – they can create serious mistakes which lead to threats to a patient's condition 2. Cultural and personal competencies go hand in hand–understand the patient from his or her own context, not yours. Understanding bring change in one's own views. 3. Implementing cultural competence in one's own ward–a challenge unless the leader of the area is on board or more than one staff member is making changes.

Pimentel et al. (25)	Colombia Community health	Explored motivational reasons for engaging with traditional medicine after participation in a five-month programme Qualitative descriptive study-written narratives	Total *n* = 4; F *n* = 2; medical students Age range 22–24 yrs Ethnicity not stated	Not defined. Referred to Kurtz et al. (62).	Medical students were motivated to explore traditional medicine because they: 1. Appreciated the structured learning format of their programme of learning because it provided a way to organize the new knowledge 2. Liked learning with and from other people rather than training them 3. Enjoyed the shared ownership with the community of the co-designed programme for children 4. Had experienced a lack training in cultural safety at university 5. Remembered prior childhood exposure to and experiences of traditional medicine
Pool (34)	America Medical-surgical oncology inpatient and outpatient	Described cancer care nurses' perspectives of the meaning of the American Indian patient–cancer care nurse relationship Interpretive phenomenological study-multiple exploratory interviews	Total *n* = 9; F *n* = 9 Age 25–71 yrs Ethnicity *n* = 6 white, *n* = 1 native Hawaiian or Pacific Islander, *n* = 2 American Indian or Alaska Native	Did not define cultural safety as aim of the study was exploring nurse-patient relationships. Referred to both Canada and New Zealand's nursing definitions of cultural safety. The Canadian document was unable to be found online (63).	Seven meta themes identified (i) task to connection–relationship is paramount, (ii) unnerving messaging–relationships do not develop because of an inability to read verbal and non-verbal patient cues, (iii) we are one–recognition by nurse that patient is a person deepens relationship and facilitates reciprocity within the relationship, (iv) the freedom of unconditional acceptance–relationships are facilitated through removal of bias, judgement and assumption, (v) attuning and opening–speaking less and listening more, (vi) atoning for the past, one moment at a time–relationship provides opportunity to honor struggles of past, show respect and reverence, (vii) humanizing the inhumane–relationship provides a link between cancer care and worlds of the patient.

Racine et al. (28)	Western Canada International placement for nursing practicum	Explored the students experience of international placements in developing cultural safety Qualitative descriptive study–semi-structured interviews	Total *n* = 7; F *n* = 6; former undergraduate nursing students Mean age at practicum, 24.6 yrs old Ethnicity *n* = 4 Canadian, others not stated	The introspective and reflexive process by which nurses examine their “biases, attitudes, stereotypes that may affect the quality of care provided to patients from ethnocultural groups” (7) (p. 13)	Three themes of (i) cultural knowledge and self-knowledge–need to be openminded about own cultural and racial biases, treat people with respect, have a willingness to learn and listen, and not being afraid of encountering cultural differences, (ii) othering–the desire to see, be exposed to and observe cultural diversity and how others manage their health systems, (iii) consciousness of neo-colonialism–being aware of issues of patriarchy, power, gender and neo-colonial relations experienced during the placements. Students learnt about themselves and others
Withall et al. (27)	Australia Rural, remote and urban locations	Explored the impact of cultural safety training on participants' practice, to what extent they incorporated cultural safety principles into their practice and identified barriers and enablers for practicing in a culturally safe manner in the work place Qualitative study – semi-structured interviews	Total *n* = 10; *n* = 7 nurses and *n* = 3 midwives trained at Flinders University in Adelaide Age not stated Ethnicity not stated	The effective care of a person/family by a health professional who has undertaken a process of reflection on their own cultural identity and recognizes the impact of their own culture on their practice (64).	Participants could theoretically define cultural safety as being centered on respect and incorporating cultural values into care. However, some participants had difficulty in describing how they incorporated principles into practice. Cultural safety was described as “treat everyone the same” and a resistance was evidenced in changing personal behavior to incorporate cultural safety principles. Others were able to describe differing needs of patients and of the barriers that existed to providing culturally safe care, and how they ensured they provided care that reflected cultural safety principles. Participants identified institutional barriers to providing culturally safe practice of limited time, resources and organizational policies, and negative attitudes of some staff.

*F, female; N, number; T1, time one; T2, time two; Trans, transgender; UK, United Kingdom; Yr, year*.

#### Stage 5: Collating, summarizing and reporting results

Twelve qualitative studies (*n* = 206 participants) were included. A broad approach to the definition of health professional was adopted and studies included participants who were nurses, nurse practitioners, midwives, dieticians, social workers, doctors and medical students, interpreters, psychologists and psychology students, occupational therapists and mental healthcare providers. Studies were undertaken between 2005 and 2021. Five studies were undertaken in Australia, two each in Canada, America, and New Zealand, and one each in Denmark and Colombia. All studies used a qualitative approach for data collection and analysis. Five studies did not report ethnicity of participants. “Cultural safety” was defined by four studies and these referred to New Zealand Nursing and Medical Council definitions or literature derived from New Zealand (24–27). The remaining nine studies referred indirectly to cultural safety (4 studies), (28–31) to cultural competence (2 studies) (32, 33) or other literature (2 studies) (34, 35). Only one study offered a definition of how the authors understood the term “culture” (24) and all operationalized the notion of culture as related to race or ethnicity.

Our approach to thematic analysis has been to take a broadly realist perspective that has assumed that people's words can represent experience in an uncomplicated manner. The analysis resulted in three themes of: (i) learning through and from direct experience, (ii) individual qualities of health professionals and, (iii) cultural training. The themes, their supporting subthemes and direct quotes are presented below (Table 3) with a narrative of the findings provided in the next section.

**Table 3:** Summary of themes and sub-themes.

**Themes**	**Sub-themes**
Learning through and from direct experience	Exposure to experiences that change the way you think and feel
Individual qualities of healthcare professionals	Intentional engagement with one's own and others values, attitudes and biases
	Developing confidence in oneself
	Practicing the skills of being in relationship
Cultural training	Content
	Process

### Stage 6: Consultation

The final stage recommended for a scoping review is consultation. Initial discussions have occurred and will continue with university teaching colleagues about scaffolding and embedding successful and supportive learning opportunities across the pre- and post- nursing registration programmes that are culturally appropriate for the local context. This review provides an evidence-based platform for wider discussions beyond our (the authors) university department. It is also timely and relevant given recent university developments. Discussion and further progress will be guided by relevant university policy documents and the Office of Māori Development.

## Findings

### Learning through and from direct experience

This theme centers around the notion that “true understanding [comes] only through experience” (31) that actively engaging with a community of people who are culturally different to oneself offers opportunities to change how a health professional makes sense of notions of culture. Experiences that can transform how someone thinks about things could take varying forms. For this medical student it was an experience of mutual learning with a community;

“They learned as much as we learned. This is not typical in research, where the researcher usually has way more knowledge and is, therefore, better able to intervene and teach. We experienced research based on participation and dialogue with the community and ourselves at the same level, both contributing to knowledge to solve problems.” (25).

There was often discomfort associated with learning through experience;

“…I learned to open up to different cultures by witnessing intolerant behaviors. I become aware, thanks to racist and xenophobic acts…that makes us think, and that helps us move forward….” (32).

This discomfort appeared to be a necessary part of the learning process;

“That moment was uncomfortable and disconcerting; we realised we came to the community to develop this project with very little knowledge about traditional medicine. However, the children taught us many things we did not know.” (25).

As well as the potential for discomfort that can come with learning through experience, there is likely a temporal element. This health professional suggests learning through experience requires a person to spend a lot of time with a community if learning is to be more than theory;

“…A lot of stuff you can't learn academically, though. Like living with them, being round another culture a lot; that is where you pick up the most, I think. Yeah, I don't know that academic learning in itself is enough…people can just put it into a little corner of their mind.” (24).

Experiences with the potential to change how a health professional thinks about a culture other than their own would seem to need time and potential discomfort to be helpful to learning. Engaging actively with a minority community appears to provide people with powerful experiential learning opportunities that have the capacity to change ways of thinking and therefore the potential to change practice.

### Individual qualities of health professionals

Significant within the results of these 12 studies are people's descriptions of what we describe as the individual qualities of health professionals that appear to allow them to practice in a culturally safe manner. These qualities can be further sub-divided into the ability to intentionally engage with one's own and others' biases and values, and the ability to use relational skills to build relationships with others. For some, this capacity for intentional engagement is aided by their own experiences of belonging to a cultural minority;

“Well, I think it's the fact that I'm from another culture that has helped me most in being open-minded because I became aware of difference and language barriers when I was very young.” (32).

For this indigenous nurse it is perhaps the experience of being part of cultural minority in tandem with an understanding of the sociopolitical influences upon health that generate a forthrightness;

“There was a gentleman who was maybe 350 kilos, the patriarch of his whānau [family], end stage everything….and one of the nurses comes up to me and says, “Well, maybe if he had a few more salads” and I just flipped…I said, “do you see that the condition that he's in, is perhaps a result of in context of a whole lot of things, not just what he puts in his mouth?”” (26).

While we cannot know from this quote the emotional aspects of this interaction, for this next health professional there is clearly something effortful about intentionally engaging with their own biases;

“…So it was alright in one way [to mix with Māori] but underneath it all the prejudices were very strong and unfortunately this was the exact opposite from what I now believe in and unfortunately those prejudices are still there and I constantly have to fight them so I am trying very hard to be conscious of it because it does go against my beliefs and I know they are there…” (24).

For those health professionals who are part of the white majority in a society, some form of emotional discomfort may be a necessary aspect of intentional engagement;

“I think we need to engage in understanding the historical relationship. As uncomfortable as colonial relations are, you need to understand them. And to be self-aware that are a white individual going into a majority African community. The only way to understand these implications is to have discussions with people who have these experiences [of colonization]” (28).

Engaging with others to understand their experiences would seem to take the capacity to tolerate one's own discomfort and to have the skills of relationship building, in particular skills of empathy, validation and curiosity about the experience of others. While it's not clear for this person that having empathy improved the quality of their relationships with the people they are caring for, it none the less changed how they related to themselves.

“when you realise whatever it took to get down here [for treatment] or what they've been though before, it's humbling..” (34).

The capacity to validate another's experience, particularly their experience of the harm done to them through colonization is viewed by this person as central to culturally safe practice;

“They [health professionals] need to get to the foundation of what it means [to provide cultural safety] and it's not about the dos and don'ts. It's actually understanding who you are and respecting people and recognizing difference and growing in yourself and acknowledging the pain and the journey that people in this country have gone through and acknowledging what's happened to [Aboriginal] people.” (30).

Curiosity and openness for this health professional allowed them to enact culturally safe practice;

“I think my listening, my ability to care for her at several different times…we [eventually] connected because of my openness in wanting to learn, and then wanting to care for her however she wanted to be cared for.” (34).

For some health professionals, it is not just words that allow them to build relationships of respect, but also the capacity to be aware of how they use their body to communicate;

“I rang the doctor; he came in, saw the patient was Māori, and just instantly changed his body language, and was more relaxed and didn't stand over him, like most other people just stand over him and talk down to him…I could see him try and build a good relationship to build trust in order to assess his injuries properly, so I was quite impressed with him. I could see in his body language what he was doing.” (26).

While a health professional's relational skills may aid their ability to practice in a culturally safe manner, it is perhaps having the confidence to act in a culturally appropriate and relationally focused way that needs to come first;

“We are taught very clearly what our boundaries are, and I was an older nurse when I did my training, and I remember going onto the ward and struggling actually because there would be Māori patients who would come up to give me a hug and I would back away, and say, ‘look I'm sorry, I'm not allowed to do that’, and I struggled with that initially, and it wasn't until a few years later when I felt confident in my own clinical practice that I actually allowed myself to be able to respond to Māori patients who came up to me to greet me.” (26).

For this indigenous nurse, their individual abilities have been unhelpfully shaped by the institutional and professional culture in which they learnt to be a nurse. People bring to their profession skills and abilities that their training needs to enhance and develop rather than stifle. Building health professional's relational skills and their ability to tolerate emotional discomfort would appear to be necessary for culturally safe practice. When these skills are put alongside the knowledge of sociopolitical influences upon health, health professionals would be well placed to engage intentionally with diversity.

### Cultural training

Cultural training initiatives have become central to efforts to change health professional practice. Barriers identified by the health professionals in these 12 studies focused on the over estimation of the role of knowledge to change practice, while enablers of helpful training experiences addressed sub-themes of process and content.

While knowledge was seen as a necessary part of training, it is not enough on its own;

“[k]knowledge is not sufficient enough to be culturally competent” (35).

Knowledge also needs to be linked to its practical application;

“I just found a lot of the time they'd just say ‘oh you need to be culturally sensitive’. Full stop. Oh yeah, great, okay, how do I do that?” (31).

What people did identify as helpful aspects of cultural training can be sub-divided into issues related to the content (knowledge or skills) being taught, and the process or way of teaching the content.

This nurse in an Australian hospital providing care to high numbers of indigenous people, talks about content that focuses on skills in addressing racism;

“[w]ell, you can see the institutional racism…the overt and covert racism that exists, and you can hear it in handover or you can hear it in the tea room…[indigenous cultural training] should help you to be able to say to people, ‘Hey, that's a really racist thing that you've said’, or ‘Why are you saying such terrible things about that particular patient?’.” (31).

Downing and Kowal (31) go on to argue that in order to address racism, knowledge of the social, political and historical context of the indigenous population is therefore essential in any training endeavor.

The process of teaching also required collaboration and learning to be situated in the practice context. A study that evaluated the impact of a cultural training programme on health professionals in a Danish university hospital found that it made a particular difference to learning;

“The ethnic patient coordinator program comprised teaching and constructive discussions based on clinical cases and patient narratives that the coordinators had themselves experienced. This situated learning made the teaching practically relevant for the coordinators and they all learned to be reflective about how to be culturally aware, sensitive and competent in specific settings and special cases.” (33).

The same study identified that designing the training in collaboration with those health professionals who were to be the “learners” was another enabling factor;

“The ethnic patient coordinator program was developed through a ‘bottom up’ process, where participants identified and defined objective and then sought ways of achieving them. This gave the coordinator's a sense of ownership while developing the program and a responsibility for spreading the approach to colleagues.” (33).

A cultural training programme that focuses on content such as racism, discrimination and colonization is not going to always be a comfortable learning environment as this is not easy subject matter:

“Cultural contexts are complex and a lot of support and assistance provided comes from building rapport, understanding one's own historical context…” (35).

With this complexity, comes discomforting emotions;

“…I find it difficult to move beyond my privilege and guilt I feel for the part of historical events occurring in Australia the negatively impacted upon Australian Aboriginals.” (35).

These two quotes come from a study that explored the nature of critical reflexivity of health professional students while taking part in an Indigenous and cross psychology paper at an Australian university. The authors, Castell et al. (35) conclude that if cultural learning experiences are to develop a person's capacity for critical reflection then the learning space must be able to tolerate and work with discomfort;

“If discomfort is a mechanism of transformative learning, it is counterproductive to elevate safety at the expense of discomfort.” (35).

In the Danish study, assisting people to work with and manage their uncomfortable feelings appears to have been achieved through the sense of support gained from being a member of a collective;

“Taking part in the ethnic coordinators team has given me the courage to take care of patients with different ethnic backgrounds. It has given me courage to focus on the patient, and I far more open to different solutions. (Field notes, midwife).” (33).

This membership of a collective of learners was also key to keeping people engaged;

“Our coordinators reported that the opportunity to work with highly engaged colleagues in the ethnic coordinators team was a significant factor in keeping everyone in the team motivated and focused on their roles and work.” (33).

From what these health professionals (and the authors of the studies within which their words appear) have to say the content of cultural training needs to directly address issues of racism and discrimination and be focused on the application of ideas into practice. Training based processes that appear to aid this include training that has been developed collaboratively with participants, training that builds a sense of community and support between learners and a training environment that expects and supports people to experience and manage feelings of discomfort.

## Discussion

This scoping review has sought to understand the health professional perspective on the barriers and enablers to them becoming culturally safe professionals. What we have found from reviewing these 12 studies is that learning experiences are at their best when they are based within communities; minority communities and collegial communities of learning. Formal trainings can incorporate the development of a community of learning as one way to move their focus beyond the simple acquisition of knowledge to learning through relationship with others. Because the ability to engage intentionally with one's own and other people's biases also seems part of the process of becoming a culturally safe health practitioner, the learning content will inevitably need to include the social, political and historical context of different cultural contexts and this in turn will likely generate emotional responses of discomfort. The learning context and those facilitating it would therefore need to have the skills to work with the emotional discomfort of learners and themselves in order to take up all opportunities for transformational learning.

There is of course a limit to what can be inferred from the reported speech of others. Inevitably we read and look for the ideas that fit with our own construction of the world. Two of the authors identify as white, one Pākehā (a New Zealander of settler origin) and the other of Welsh heritage. The third identifies as Kāi Tahu (the largest iwi (tribe) of the South Island of New Zealand). All three are nurses and educators. Therein lies one of the strengths of this review, which is the indigenous oversight provided to the project. There are a number of limitations to this review. The first is inherent within the nature of scoping reviews and the expectation that data can be neatly grouped and synthesized as discrete entities (36). These findings, while written up as if separate themes, can also be seen to hold overlapping content. We consider this the nature of the data rather than a lack of synthesis but this is a view that may not be shared by all readers. Secondly there would appear to be limited published data available currently that helps us to understand the health professional perspective on what helps them to become culturally safe practitioners. This may reflect our search strategy. We used the terms “clinician” and “health professional” as our focus was on understanding the experience of clinicians in practice. While the student perspective was not excluded as evidenced by the use of the article from Colombia, it is possible that this choice limited the data available to us our search did not identify a similar article from Canada (37). We also did not use the term “cultural humility” and as a result may have missed any articles that only use this term to explain constructs similar to cultural safety and neither did we breakdown the term “health professional” to name all the different professions. By choosing to focus on the development of cultural safety by those who engage with the construct of cultural safety, this review does not include the perspectives of those who do not. It also does not address in depth the evident tensions between how professionals are trained to construct boundaries in relationships alongside the need for more human connection. None the less, these findings still allow us to speculate on how we might go about building a workforce of health professionals who can work effectively with people who have long-term conditions and who are also from unique and diverse communities.

Culture shapes how we think about notions of illness, suffering and wellbeing. It shapes our sense of self, who we are as a person and are ideas about how relationships operate. It also influences our access to social resources such as employment, health and education services. It provides us with the assumed responses to questions such as “Is what I am experiencing an ‘illness’?,” “Who, if anyone, should I turn to for help if I think of myself as ill?.” Culture shapes how a person makes sense of themselves as the “patient” and as the “health professional.” Cultural considerations are therefore central to how a person lives with a health condition that cannot be “cured,” and is therefore understood by the health system to require access to care that will support ongoing self-management (5).

Self-management concepts are strongly driven by ideas of personal responsibility and an expectation that a person will learn to manage themselves. This too is a cultural artifact, a way of thinking that comes from white, Western, neoliberal countries (38, 39). This means that while it is a very dominant way of thinking, it is not the only way to think. A health practitioner who is able to practice in a culturally safe way will be better able to recognize and work with these issues. This higher quality health care increases the chances of improved outcomes for people.

As educators, our (the authors) interest is with how to provide learning environments that are more likely to build a workforce of culturally safe health professionals. While this review does not provide us with definitive answers, it allows us to speculate about the importance of becoming part of communities of learning, of developing health professionals' relational skills, of attending to both process and content in the classroom and being able to tolerate and work with emotional discomfort.

### Learning through and from direct experience

We found health professionals spoke about the important learning that came from a sense that they were part of a minority community, that it exposed them to different world views and to work successfully in the environment they were compelled to find ways to practice that “fitted” with the community. This was particularly noted when health professionals engaged in a mutual learning experience with a minority community. While not limited to learning that happens outside of a classroom, these experiences of learning from being part of a minority community seem highly likely to generate experiences named as the “disorientating dilemma” which Transformative Learning Theory posits is the first stage in how adults become aware of their hitherto unquestioned values and beliefs, critically reflect on these and then change or revise how they make sense of the world. While there remains debate about whether transformative learning is a distinct theory or simply a metaphor to make sense of adult learning, these are useful ideas to draw on when thinking about how to create learning environments that have the potential to change people's ideas about themselves and others (40, 41).

While becoming part of a minority community may provide important learning opportunities for health professionals, it is not something to be undertaken lightly. Minority communities are routinely exposed to the impacts of power imbalances and it is health professionals' lack of attention to these dynamics that arguably perpetuates these problems. The values that guide health professional practice are not necessarily the same as or even similar to as the minority community which they work within or hope to become a part (42). As experienced by Oosman et al. (37) the success of a student physiotherapy practicum within an indigenous community was made possible because of the attention paid to collaborative relationships between all parties and the value placed upon making the wishes of the community paramount in all decisions.

### Individual qualities of health professionals

This review suggests that one of the qualities required of individual health practitioner is the capacity to engage intentionally with the experience of another person. This is a central relational skill for us all, and is particularly important when seeking to build a relationship with someone whose experience of the world we can recognize immediately as different from our own. From the work of John Bowlby, Mary Main and Mary Ainsworth and the development of attachment theory, we have a good understanding of how an adult's capacity to be in relationship with others is shaped by their experience of relationships with their caregivers when they were a child. A caregiver's capacity to use skills of empathy, validation and curiosity shapes the development of a secure attachment style which in turn provides an adaptive relational template for the adult (43). An adult with a secure attachment style is more able to know and regulate their own emotions, recognize that each person's actions are driven by their own mental state and respond intuitively to others emotional states (44, 45). While ideally these are skills we learn from childhood, they are not static qualities and we can all develop and enhance these skills as a means to improve the quality of our adult relationships. These skills, while central to the work of mental health professionals, are arguably vital for all health professionals who want to engage holistically with the people to whom they provide care.

### Cultural training

Through-out these articles there is evidence that when creating potentially transformative learning environments we need to attend to both issues of content and process; that it is not just the particular subject matter or knowledge that needs to be taught e.g., the historical and political context of a minority community, but it is also the process or the how of learning. For example, one of the key learning processes that can be seen is how learning takes place through relationships with colleagues which suggests that educators need to factor this in to the design the experience. Llerena-Quinn (46) describes this need to attend to issues of process in her reflection on providing a cultural awareness course to colleagues at a medical school. Even when the primary goal of the course was to increase people's level of comfort with talking about complex issues of difference, it was easy to become distracted by discussions related to skills and “cases.” They also identified ways they could improve the structure of the course to create the “holding environment” needed to allow people to engage with their discomfort.

Creating a learning environment that is able to pay attention to how people learn within a group would also seem to be an element of process that needs to be attended to. Within mental health nursing and other mental health related disciplines, the use of clinical supervision to support learning and professional development is well established. Looking for opportunities to create a sense of learning as part of a group is not dissimilar from the intent of clinical supervision (47). The value of groups as an environment in which we learn about ourselves is also a fundamental aspect of group psychotherapy (48). Process is also an important consideration when thinking about culturally safety as being something that develops for a health professional over time. While a one-off training might provide the “disorientating dilemma” and the sense of safety needed to examine one's values and beliefs, this in itself is unlikely to bring forth a fully-fledged, culturally safe practitioner. As argued by Molloy et al. (29) if cultural safety is seen as a process where by people move through phases, then educational environments need to factor in how they develop a sense of community within their learners so that people are able to develop mutually supportive relationships over the long term that can provide a place to reflect on experience, ask questions and seek guidance.

### A pedagogy of discomfort

Each of these elements draws on in some way the issue of discomfort. Throughout our findings are references to how health professionals experienced feelings of discomfort. To again draw on ideas about transformational learning environments, a “disorientating dilemma” by its nature is likely to generate unsettling emotions and ideas from attachment theory would suggest that not all people come well equipped to be aware of and to tolerate these.

Boler and Zembylas (49) and Zembylas (50) argue for higher education teaching practices to be based upon a pedagogy of discomfort; teaching practices that expect and support students to move outside of their emotional “comfort zones.”

“*This approach is grounded in the assumption that discomforting feelings are important in challenging dominant beliefs, social habits and normative practices that sustain social inequities and they create openings for individual and social transformation” [(**50**)* *p. 8]*.

Enacting this approach to teaching practice requires educators to have the relational skills to create a learning environment that values the emotional world of students which could present itself in the form of tearfulness, anger, irritability, and withdrawal into silence. This in turn requires the educator to have their own skills in empathy, validation and curiosity and be able to tolerate their own emotional discomfort. We note anecdotally that this does not necessarily form part of how adult educators are trained and as with the process of becoming culturally safe, it is likely a process that requires space for ongoing support and reflection in order to develop oneself as a teacher in this way.

### Further research

The lack of information available in the literature to help us understand the health professional perspective on what has helped and hindered them in becoming culturally safe practitioner has compelled us to write in a speculative way. While this lack of information may in part be attributed to the identified limitations of our chosen search strategy, there would none-the-less seem to be a significant gap in the literature which lead us to conclude that it is an area ripe for further research.

## Conclusion

There is a dearth of information that explores professionals' perspectives on what helps them to translate cultural safety concepts into practice. From what we have been able to find, we have speculated that when designing learning environments, health professional educators need to pay attention to issues of both content and process and the latter is probably the aspect that gets most often missed. Culturally safe practitioners are able to recognize their own values and biases, be flexible and open minded about the experiences of others and are able to recognize how historical and social processes have impacted upon people's experience of health and health care services. This review suggests that if we are to grow a workforce with these abilities then we could pay more attention to how people learn from direct experience and from being part of a community of learners. It also suggests that those facilitating the learning environment need to pay attention to the learning opportunities that are made available by teaching practices that enfold a tolerance of discomfort.

That said, there is a range of literature not necessarily referred to here, that explores the theory and practice of how to facilitate learning environments that promote an adult's capacity to examine and review their values and beliefs. Many authors agree that we need to train health professionals to be culturally safe and that it is essential we do so if we are to address inequities in health, particularly for those people from unique and diverse communities. Likewise, there are also a number of authors who consider how best to train health professionals to be culturally competent and safe practitioners but there are very few examples of health professionals speaking from their own experiences about what helps and what does not. Theory is all very well but the challenge is how to translate this into practice. That is where the health professional perspective is a potential untapped resource to improve health care for people with long-term conditions from unique and diverse populations.

## Data availability statement

The original contributions presented in the study are included in the article/supplementary material, further inquiries can be directed to the corresponding author.

## Author contributions

Conceptualization: LW. Data curation, formal analysis, methodology, and writing original draft: LW and AW. Writing—review and editing: all authors. All authors have approved the final manuscript.

## Funding

Publication of this work was supported by the Centre for Postgraduate Nursing Studies.

## Conflict of interest

The authors declare that the research was conducted in the absence of any commercial or financial relationships that could be construed as a potential conflict of interest.

## Publisher's note

All claims expressed in this article are solely those of the authors and do not necessarily represent those of their affiliated organizations, or those of the publisher, the editors and the reviewers. Any product that may be evaluated in this article, or claim that may be made by its manufacturer, is not guaranteed or endorsed by the publisher.
